# Association Between COVID-19 and Planned and Postponed Cancer Screenings Among American Indian Adults Residing in California and Oklahoma, March–December 2020

**DOI:** 10.1177/00333549241254226

**Published:** 2024-06-04

**Authors:** Julie H.T. Dang, Sixia Chen, Spencer Hall, Janis E. Campbell, Moon S. Chen, Mark P. Doescher

**Affiliations:** 1 Department of Public Health Sciences, Division of Health Policy and Management, University of California, Davis School of Medicine, Sacramento, CA, USA; 2 Department of Biostatistics and Epidemiology, Hudson College of Public Health, University of Oklahoma Health Sciences Center, Oklahoma City, OK, USA; 3 Department of Internal Medicine, Division of Hematology and Oncology, University of California, Davis School of Medicine, Sacramento, CA, USA; 4 Stephenson Cancer Center, College of Medicine, Department of Family and Preventive Medicine, University of Oklahoma Health Sciences Center, Oklahoma City, OK, USA

**Keywords:** American Indian, COVID-19, cancer screening

## Abstract

**Objective:**

Little is known about how the COVID-19 pandemic affected cancer screenings among American Indian people residing in California and Oklahoma, 2 states with the largest American Indian populations. We assessed rates and factors associated with cancer screenings among American Indian adults during the pandemic.

**Methods:**

From October 2020 through January 2021, we surveyed 767 American Indian adults residing in California and Oklahoma. We asked participants whether they had planned to obtain screenings for breast cancer, cervical cancer, and colorectal cancer (CRC) from March through December 2020 and whether screening was postponed because of COVID-19. We calculated adjusted odds ratios (AORs) for factors associated with reasons for planned and postponed cancer screening.

**Results:**

Among 395 participants eligible for breast cancer screening, 234 (59.2%) planned to obtain the screening, 127 (54.3%) of whom postponed it. Among 517 participants eligible for cervical cancer screening, 357 (69.1%) planned to obtain the screening, 115 (32.2%) of whom postponed it. Among 454 participants eligible for CRC screening, 282 (62.1%) planned to obtain CRC screening, 80 of whom (28.4%) postponed it. In multivariate analyses, women who lived with a child (vs did not) had lower odds of planning to obtain a breast cancer screening (AOR = 0.6; 95% CI, 0.3-1.0). Adherence to social distancing recommendations was associated with planning to have and postponement of cervical cancer screening (AOR = 7.3; 95% CI, 0.9-58.9). Participants who received (vs did not receive) social or financial support had higher odds of planning to have CRC screening (AOR = 2.0; 95% CI, 1.1-3.9).

**Conclusion:**

The COVID-19 pandemic impeded completion of cancer screenings among American Indian adults. Interventions are needed to increase the intent to receive evidence-based cancer screenings among eligible American Indian adults.

During the COVID-19 pandemic, federal agencies and professional medical societies recommended postponement of preventive care visits to prioritize health care resources.^1-3^ In the United States, approximately 9.4 million screenings for breast, colorectal, and prostate cancer did not occur because of the COVID-19 pandemic.^4^ Timely cancer screening is essential to primary and secondary prevention of cancer.^5,6^

Although rates of cancer screening and cancer diagnosis have improved since the initial phase of the COVID-19 pandemic, models predicted increased rates of advanced-stage cancer at the time of diagnosis and increased rates of morbidity and mortality because of decreased and delayed cancer screening in 2020.^7-9^ Delayed cancer screenings can exacerbate disparities among American Indian and Alaska Native (AI/AN) people, who have significantly higher rates of cancer incidence and mortality compared with non-Hispanic White people.^10-14^ Depending on geographic region and source, AI/AN people are more likely than non-Hispanic White people to acquire and die from cancers for which screenings exist.^10-16^ Studies documented sharp declines in the volume of screening for breast cancer, cervical cancer, and colorectal cancer (CRC) among AI/AN people compared with non-Hispanic White people during the COVID-19 pandemic.^17-19^

In this study, we used survey data to examine rates of screening for breast cancer, cervical cancer, and CRC during the COVID-19 pandemic among AI/AN adults residing in California and Oklahoma, 2 states with the largest AI/AN populations.^20^ We aimed to assess factors associated with plans among AI/AN adults to obtain screening for breast cancer, cervical cancer, and CRC during the COVID-19 pandemic and whether those plans were postponed because of the pandemic. We also analyzed rates of planned and postponed cancer screenings among survey participants. The term American Indian (AI) rather than AI/AN is used throughout the remainder of this article in reference to our sample population, because few Alaska Native people reside in California and Oklahoma.

## Methods

### Sample Selection and Data Collection

We used survey responses from a subset of survey questions developed by the National Cancer Institute–funded Impact on COVID-19 on the Cancer Continuum Consortium (IC-4) to assess the effects of COVID-19 on cancer prevention, control, and survivorship. The University of California, Davis (UCD) Comprehensive Cancer Center, and the University of Oklahoma Stephenson Cancer Center (OUSC) administered the survey to 1068 AI adults. From this sample, we selected 3 distinct cohorts of participants to assess planned and postponed cancer screenings that included mammograms for breast cancer screening (women aged 40-74 y), Papanicolaou (Pap) tests for cervical cancer screening (women aged 21-65 y), and colonoscopy and stool blood tests (men and women aged 50-75 y) for CRC screening. Respondents answered whether they had planned to have a specific cancer screening test conducted from March through December 2020 (yes or no); if respondents answered yes to that question, the follow-up question was whether they or their doctor postponed the test because of COVID-19 (yes or no). UCD and OUSC administered the survey from October 2020 through January 2021.

### Covariates

Although the complete survey consisted of 58 core questions, this study reported on the 30 questions related to sociodemographic characteristics, COVID-19–related attitudes and behaviors, and cancer screening behaviors. The survey questions included sociodemographic variables found to be predictors of cancer screenings among racially and ethnically diverse populations.^21-23^ The survey questions on health behaviors related to COVID-19 were based on the Health Belief Framework.^24^ The Health Belief Framework posits that individuals are more or less likely to engage in preventive behaviors based on their perceived susceptibility to and severity of the sickness or disease. The COVID-19 questions aimed to assess whether individuals engaged in preventive measures to reduce their risk of getting COVID-19.^25^

### Sociodemographic Variables

On the survey, respondents indicated their age group (18-39, 40-59, or ≥60 y), sex (female or male), Hispanic ethnicity (yes or no), educational attainment (high school diploma/General Educational Development or ≥some college), marital status (single/never married, married/living together, or separated/divorced/widowed), lived in a house with a child (eg, a person aged <18 y; yes or no), annual household income (<$35 000 or ≥$35 000), covered by private or public health insurance or another kind of health plan (Indian Health Service; yes or no), had a preexisting condition (the survey provided a list of conditions and a fill-in-the-blank option), general health status (excellent or good, fair or poor), and employment status before COVID-19 (full-time/part-time employment or unemployed/other occupation). The other occupational categories included students, retired people, homemakers, and people with disabilities.

### COVID-19 Variables

The survey presented a list of social distancing activities, and respondents answered whether they performed the following activities all or most of the time (yes or no): staying at home except for going to work, outdoors to exercise, to the grocery store, to the pharmacy, or to get medical care; not having anyone come into their home; staying 6 feet away from people when they leave the house; wearing a face mask when outdoors; and wearing a face mask when inside a place besides their home. Respondents also answered questions on the perceived importance of social distancing recommendations (very important to somewhat important or a little important to not important) and whether they had ever been tested for COVID-19 (yes or no); had been in close physical contact with a person with a positive COVID-19 test in the past 30 days (yes or no); had attended any gatherings, rallies, demonstrations, or other social gatherings with >2 people outside their household (yes or no); and had received support (eg, emotional, materials, financial) during the pandemic from family or friends.

### Statistical Analysis

We expressed descriptive statistics as counts and percentages. We calculated the proportion of survey respondents who were eligible (according to age and sex) for each cancer screening test. We then calculated the proportion of respondents who had planned to obtain the cancer screening and who had planned to obtain the cancer screening but reported that screening was postponed.

We examined binary associations between covariate variables and outcome variables by using the Pearson χ^2^ test or the Fisher exact test when the expected cell counts of some cells were <5. We conducted multivariate analysis between outcome variables and covariate variables by using multivariate logistic regression. We used stepwise model selection to select the predictors. First, we conducted a binary analysis to identify the list of significant predictors in each model. Second, we examined collinearity among the list of significant predictors. For our analysis, we retained predictors among the group of mutually correlated predictors with a correlation coefficient >0.6. Finally, we used stepwise model selection to select the final model, with *P* < .05 considered significant. We also checked for confounding and interaction between the predictors in the model. For CRC screening, we combined stool-based screening tests and colonoscopies into 1 outcome variable because of the small sample size. We included covariate variables in the final model for each outcome variable. We determined the adjusted odds ratios (AORs) for associations between dependent (ie, outcome) variables and independent (ie, predictor) variables. We excluded respondents with missing outcome values from the bivariate and multivariate analyses. We used SAS version 9.4 (SAS Institute Inc) for statistical analysis.

The university institutional review boards (IRBs) at UCD (IRB no. 1639547-4) and OUSC (IRB no. 12190) approved the study; in Oklahoma, the Choctaw Nation (IRB no. 2020-003) and Cherokee Nation (IRB no. 338) IRBs also approved the study. All survey participants gave written or verbal consent.

## Results

The overall sample included 767 AIs who were eligible for breast cancer, cervical cancer, and/or CRC screenings. Among respondents, 395 women were eligible for mammography, 517 women were eligible for Pap testing, and 454 people were eligible for stool-based screening and/or colonoscopies (Table 1). Of 395 women eligible for mammograms, 234 (59.2%) responded that they were planning to obtain one; however, 127 of 234 (54.3%) postponed mammograms because of COVID-19. Of 517 women eligible for Pap testing, 357 (69.1%) had planned to obtain one; however, 115 of 357 (32.2%) postponed Pap tests because of COVID-19. Of 454 adults eligible for CRC screening, 282 (62.1%) responded they were planning to obtain one; however, 80 of 282 (28.4%) postponed CRC screening because of COVID-19.

**Table 1.** Demographic characteristics and COVID-19 safety behaviors and attitudes of American Indian adults residing in California and Oklahoma who were eligible for breast, cervical, and/or colorectal cancer screening, March through December 2020^a^

Characteristic	No. (%) of participants
Residence (n = 767)	
Oklahoma	453 (59.1)
California	314 (40.9)
Age group, y (n = 760)	
18-39	219 (28.8)
40-59	270 (35.5)
≥60	271 (35.7)
Sex (n = 765)	
Female	622 (81.3)
Male	143 (18.7)
Hispanic (n = 673)	
No	564 (83.8)
Yes	109 (16.2)
Educational attainment (n = 762)	
High school diploma/GED	240 (31.5)
≥Some college	522 (68.5)
Annual household income (n = 695)	
<$35 000	305 (43.9)
≥$35 000	390 (56.1)
Health insurance (n = 758)	
Yes	651 (85.9)
No	107 (14.1)
Health status (n = 761)	
Excellent or good	536 (70.4)
Fair or poor	225 (29.6)
Marital status (n = 760)	
Single, never been married	105 (13.8)
Married or living together	430 (56.6)
Separated/divorced/widowed	225 (29.6)
Employment prepandemic (n = 695)	
Full- or part-time employment	305 (43.9)
Unemployed or other^b^	390 (56.1)
Child residing in household (n = 767)	
Yes	463 (60.4)
No	304 (39.6)
No. of social distancing guidelines followed (n = 713)	
≤2	89 (12.5)
≥3	624 (87.5)
Importance of social distancing during COVID-19 (n = 747)	
Very to somewhat	657 (88.0)
Little to not	90 (12.0)
Tested for COVID-19 (n = 754)	
Yes	492 (65.3)
No	262 (34.7)
Close physical contact with person who tested positive for COVID-19 (n = 706)	
Yes	135 (19.1)
No	571 (80.9)
Supported during the COVID-19 lockdown (n = 713)	
Yes	203 (28.5)
No	510 (71.5)
Attended gatherings (>2 people outside of household) (n = 767)	
Yes	327 (42.6)
No	440 (57.4)
Have a preexisting condition (other than cancer) (n = 767)	
Yes	620 (80.8)
No	147 (19.2)
Eligible for mammography and screening planned (n = 395)	
Yes	234 (59.2)
No	161 (40.8)
Mammography planned and postponed because of COVID-19 (n = 234)	
Yes	127 (54.3)
No	107 (45.7)
Eligible for Pap test and screening planned (n = 517)	
Yes	357 (69.1)
No	160 (30.9)
Pap test planned and postponed because of COVID-19 (n = 357)	
Yes	115 (32.2)
No	242 (67.8)
Eligible for colorectal cancer screening and screening planned (n = 454)	
Yes	282 (62.1)
No	172 (37.9)
Colorectal cancer screening planned and postponed because of COVID-19 (n = 282)	
Yes	80 (28.4)
No	202 (71.6)

Abbreviations: GED, General Educational Development; Pap, Papanicolaou.

a Data source: subset of survey questions developed by the National Cancer Institute–funded Impact on COVID-19 on the Cancer Continuum Consortium; participants were surveyed from October 2020 through January 2021.

b Other included student, retired, homemaker, and person with disability.

In bivariate analysis, significant predictors associated with planning to obtain a mammogram included having a preexisting condition other than cancer, health insurance status, and residing with a minor. Significant predictors associated with planning to obtain a Pap test included attending gatherings with more than 2 people outside of your household and the number of social distancing recommendations followed. The only significant predictor associated with planning to obtain a CRC screening was health insurance status (Table 2).

**Table 2.** Bivariate analysis of demographic characteristics and COVID-19 safety behaviors and attitudes among American Indian adults residing in California and Oklahoma who had planned a cancer screening, March through December 2020^a^

Characteristic	Mammography planned (women aged 40-74 y) (n = 234)		Pap test planned (women aged 21-65 y) (n = 357)		CRC screening planned (adults aged 50-75 y) (n = 282)	
	No. (%)	*P* value^b^	No. (%)	*P* value^b^	No. (%)	*P* value^b^
Age group, y		.09		.15		.08
18-39	—		196 (54.9)		—	
40-59	124 (53.0)		120 (33.6)		102 (36.2)	
≥60	105 (44.9)		36 (10.1)		180 (63.8)	
Sex		—		—		.22
Female	234 (100.0)		357 (100.0)		180 (63.8)	
Male	—		—		105 (37.2)	
Hispanic		.98		.29		.36
No	155 (66.2)		75 (21.0)		190 (67.4)	
Yes	22 (9.4)		240 (67.2)		24 (8.5)	
Educational attainment		.84		.85		.90
High school diploma/GED	104 (44.4)		113 (31.7)		129 (45.7)	
≥Some college	130 (55.6)		243 (68.1)		157 (55.7)	
Annual household income		.50		.93		.23
<$35 000	96 (41.0)		137 (38.4)		119 (42.2)	
≥$35 000	106 (45.3)		188 (52.7)		121 (42.9)	
Health insurance		.03		.28		.02
Yes	210 (89.7)		287 (80.4)		264 (93.6)	
No	21 (9.0)		66 (18.5)		19 (6.7)	
Health status		.98		.02		.44
Excellent or good	151 (64.5)		264 (73.9)		196 (69.5)	
Fair or poor	82 (35.0)		91 (25.5)		89 (31.6)	
Marital status		.46		.31		.79
Single, never been married	29 (12.4)		70 (19.6)		27 (9.6)	
Married or living together	103 (44.0)		211 (59.1)		136 (48.2)	
Separated/divorced/widowed	100 (42.7)		74 (20.7)		119 (42.2)	
Employment prepandemic		.89		.20		.46
Full-time or part-time	97 (41.5)		239 (66.9)		94 (33.3)	
Unemployed or other^c^	132 (56.4)		112 (31.4)		185 (65.6)	
Child in household		.01		.15		.51
Yes	155 (66.2)		195 (54.6)		66 (23.4)	
No	79 (33.8)		162 (45.4)		221 (78.4)	
No. of social distancing guidelines followed		.24		.42		.72
≤2	10 (4.3)		43 (12.0)		9 (3.2)	
≥3	209 (89.3)		286 (80.1)		263 (93.3)	
Importance of social distancing		.15		.34		.67
Very to somewhat	216 (92.3)		293 (82.1)		268 (95.0)	
Little to not	11 (4.7)		56 (15.7)		6 (2.1)	
Tested for COVID-19		.07		.38		.56
Yes	145 (62.0)		242 (67.8)		165 (58.5)	
No	85 (36.3)		112 (31.4)		118 (41.8)	
Attended gatherings (>2 people)		.68		.009		.57
Yes	126 (53.8)		213 (59.7)		129 (45.7)	
No	108 (46.2)		144 (40.3)		158 (56.0)	
Preexisting condition		.008		.31		.23
Yes	195 (83.3)		259 (72.5)		252 (89.4)	
No	39 (16.7)		98 (27.5)		35 (12.4)	
Supported during COVID-19 lockdown		.19		.40		.28
Yes	77 (32.9)		105 (29.4)		73 (25.9)	
No	142 (60.7)		236 (66.1)		196 (69.5)	

Abbreviations: —, does not apply; CRC, colorectal cancer; GED, General Educational Development; Pap, Papanicolaou.

a Data source: subset of survey questions developed by the National Cancer Institute–funded Impact on COVID-19 on the Cancer Continuum Consortium; participants were surveyed from October 2020 through January 2021. Percentages may not total to 100 because of missing values.

b Significant at *P* < .05 using Pearson χ^2^ test.

c Other included student, retired, homemaker, and person with disability.

Multivariate analyses showed that eligible AI women with (vs without) a preexisting condition other than cancer had higher odds of planning to obtain a mammogram (AOR = 2.3; 95% CI, 1.1-4.8) (Table 3). Women who lived with a child (vs did not live with a child) had lower odds of planning to get a mammogram (AOR = 0.6; 95% CI, 0.3-1.0). Women who had been tested for COVID-19 (vs not tested for COVID-19) had higher odds of postponing their mammogram (AOR = 5.7; 95% CI, 1.6-20.3).

**Table 3.** Multivariate regression analysis of predictors of planned and delayed cancer screenings among American Indian adults residing in California and Oklahoma, March through December 2020^a^

Factor	Adjusted odds ratio (95% CI) [*P* value^b^]					
	Mammogram planned (n = 234)	Mammogram delayed (n = 127)	Pap test planned (n = 357)	Pap test delayed (n = 115)	CRC screening planned (n = 279)	CRC screening delayed (n = 287)
Having a preexisting condition other than cancer (yes vs no)	2.3 (1.1-4.8) [.02]^b^	—	—	—	—	—
Child residing in household (yes vs no)	0.6 (0.3-1.0) [.04]^b^	—	—	—	—	—
Had a COVID-19 test (yes vs no)	—	5.7 (1.6-20.3) [.007]^b^	—	—	—	—
Health status (excellent or good vs fair or poor)	—	—	1.7 (1.0-2.9) [.06]	—	—	—
Has been in any group of >2 people outside the household (yes vs no)	—	—	1.7 (1.1-2.8) [.02]^b^	0.4 (0.2-0.9) [.03]^b^	—	—
No. of social distancing guidelines followed (≤2 vs ≥3)	—	—	—	7.3 (0.9-58.9) [.06]	—	—
Support during COVID-19 pandemic (yes vs no)	—	—	—	—	2.0 (1.1-3.9) [.03]^b^	—
Health insurance status (yes vs no)	—	—	—	—	—	7.7 (1.0-58.6) [.05]^b^

Abbreviations: CRC, colorectal cancer; Pap, Papanicolaou.

a Data source: subset of survey questions developed by the National Cancer Institute–funded Impact on COVID-19 on the Cancer Continuum Consortium; participants were surveyed from October 2020 through January 2021.

b Significant at *P* < .05 using multivariate logistic regression.

Among eligible AI women, characteristics independently associated with higher odds of planning to have a Pap test included having excellent or good (vs fair or poor) health (AOR = 1.7; 95% CI, 1.0-2.9) and attending (vs not attending) a gathering with >2 individuals from outside their household (AOR = 1.7; 95% CI, 1.1-2.8). Women who followed ≥3 (vs ≤2) social distancing guidelines had higher odds of postponing a Pap test (AOR = 7.3; 95% CI, 0.9-58.9). Women who attended (vs did not attend) a gathering with >2 people from outside their household had lower odds of postponing their Pap test (AOR = 0.4; 95% CI, 0.2-0.9).

Among eligible AI adults, those who received (vs did not receive) support during the COVID-19 pandemic had higher odds of planning to have a CRC screening (AOR = 2.0; 95% CI, 1.1-3.9). Adults with (vs without) health insurance coverage had higher odds of postponing their CRC screening (AOR = 7.7; 95% CI, 1.0-58.6).

## Discussion

According to our survey results, more than half of AI participants eligible for a cancer screening had planned to get screened, but more than one-quarter of eligible AI participants reported that their screening was postponed and/or delayed because of COVID-19. The percentage of eligible AI participants who reported having a planned cancer screening in our study was higher than reported by Dennis et al.^18^ In that study, in 2020, 35.4% of AI/AN women had a mammogram, 40.1% of AI/AN women had a Pap test, and 13.8% of AI/AN adults had a CRC screening.^18^ Further investigation is needed to assess whether people who had planned to get a cancer screening received the screening.

Rates of postponed and/or delayed cancer screenings because of COVID-19 among AI adults in our study were also higher than in a study at another IC-4 consortium site.^26^ In that study, 24.5% of women had planned and delayed a mammogram (vs 54.3% of respondents in our study), 27.1% of women had planned and delayed a Pap test (vs 32.2% of respondents in our study), and 28.3% of participants had planned and delayed CRC screening (vs 36.2% of respondents in our study).^26^ Our findings align with findings from the Centers for Disease Control and Prevention, which estimated that, by June 30, 2020, about 32% of US adults reported avoiding routine medical care because of COVID-19 concerns.^27^

Unlike other studies that examined general delays in cancer screenings, our study focused only on AI people and included data on demographic characteristics and COVID-19 behaviors.^19,28-30^ The higher rates of postponed and/or delayed cancer screenings that we found in our study compared with what has been reported for the general population are alarming as, historically, AI communities have faced additional challenges and barriers to obtaining recommended cancer screenings (eg, transportation, geographic isolation, culture, mistrust of the health care system).^31-33^ Health care organizations should consider employing patient navigators to help reduce barriers to cancer screening for AI people. Patient navigation has proven to be an effective and successful model to increase cancer screenings among underresourced (eg, high poverty and low income) and medically underserved communities.^34,35^

Of the 3 cancer screenings, our study found the highest rates of postponement among AI women eligible for mammography. The Kaiser Family Foundation reported that 49% of women reported postponing some type of medical care during the pandemic, with 23% stating that they skipped preventive screenings.^36^ Not surprisingly, in our study, the odds of having a planned mammogram were 2.3 times greater among AI women who had a preexisting condition than among AI women without a preexisting condition. Women with preexisting conditions may be more attuned to their health care needs and more vigilant of their preventive care than women without preexisting conditions, because they may have to seek continuous primary care for their other health conditions. In addition, we surmised that women who had a COVID-19 test, compared with women who did not have a COVID-19 test, would be more likely to postpone their mammogram because these women may be more concerned about being infected.

AI women who reported living with a child had 40% lower odds of planning to have a mammogram than AI women who did not live with a child. Women who live with children may have childcare responsibilities that make it more logistically challenging for them to have a planned mammography compared with women who do not live with children. The COVID-19 pandemic resulted in a greater effect on mothers than on men and women without children.^37,38^ Effects included high rates of unemployment and the additional need for childcare because schools and daycares were closed.^39-41^ In addition to their already disproportionate caretaking roles in families, many mothers assumed additional responsibilities during the COVID-19 pandemic, so they may have postponed their mammograms. In our survey, we did not ask AI women if they were mothers; data are lacking on the role of motherhood responsibilities on cancer screening practices during the COVID-19 pandemic.

The second highest rate of cancer screening postponement in our study was among AI women eligible for Pap testing; 32.2% of AI women postponed their tests because of COVID-19. Our finding is higher than the 27.1% delay in Pap screening reported by Zhang et al.^26^ Among women eligible for a Pap test, several COVID-19–related behavioral factors were associated with plans and postponements of tests. Women who attended gatherings outside their household had higher odds of planned Pap testing than women who did not have gatherings outside their household. This finding may be attributable to the positive effects of social support among women on cervical cancer screening.^42,43^ Although not a direct measure of social support, attending social gatherings can be an opportunity to obtain social support. Conversely, AI women who followed ≥3 social distancing guidelines were more likely to postpone their Pap testing than AI women who followed fewer social distancing guidelines. The role of social support networks needs to be further explored as we did not ask questions about the types of gatherings attended by AI women.

Among AI adults eligible for CRC screening, 28.4% postponed screening because of COVID-19. Zhang et al^26^ reported that 11% of participants delayed stool blood tests and 36% delayed colonoscopies during the COVID-19 pandemic.^26^ Our study showed that AI adults who received support during the pandemic had twice the odds of having a planned CRC screening than those who did not. This finding is aligned with another study that reported the positive effect of social support on mental health during the COVID-19 pandemic.^44^ Our study is the first to report on the positive effects of social support on CRC screening during the pandemic. Although social support has shown a positive effect on cancer screening during nonpandemic times,^45,46^ we need to further assess the effects of perceived and received social support during the pandemic on cancer screenings. Understanding these measures can help public health professionals and clinicians develop and plan for future interventions in times of disruptions to normal health care services. Not surprisingly, the odds of having a CRC screening were 7.7 times greater for those with health insurance than for those without health insurance, a finding consistent with previous work on predictors of CRC screening use in the United States.^47,48^

### Limitations

Our study had some limitations. First, we used a convenience sampling frame that collected cross-sectional, self-reported data. Second, we may not have been able to establish an association between cancer screening postponement and/or delay and the covariates (eg, COVID-19–related health behaviors) because the recall window for the screening questions (March–December 2020) and when the survey was administered (October 2020–January 2021) were not the same. Our findings may reflect a high level of health consciousness that is positively correlated with both social distancing and postponing and/or delaying cancer screening. Third, we did not ask why a cancer screening was postponed, whether due to health care provider or participant delay or postponement of the appointment. Fourth, we did not assess the prevalence of COVID-19 in our sample; having COVID-19 may have contributed to high rates of postponed and/or delayed cancer screenings. During the recall time frame for cancer screenings, the 7-day positivity rate for COVID-19 ranged from 0% to 14.4% in California and from 0% to 22.8% in Oklahoma.^49^ More research is needed to assess whether the prevalence of COVID-19 in the community was associated with postponing and/or delaying routine cancer screenings. Fifth, our missing data disproportionately affected the sample of Hispanic respondents; therefore, findings on this group merit caution.

Our study also had several strengths, including a large sample size of AI adults residing in California and Oklahoma and a high response rate. Response rates of ≥60% have been considered as excellent according to previous literature.^50-52^ In addition, our study is the only one that focused exclusively on AI adults during COVID-19.

## Conclusion

The effects of COVID-19 on cancer outcomes among AI adults will continue to be felt for years. Our study found high rates of postponement of cancer screenings among AI adults residing in California and Oklahoma. These delays could lead to an increase in late-stage cancer diagnosis and mortality, further exacerbating the disproportionate incidence of cancer among AI people. Cancer screening interventions and public health campaigns are needed to ensure that prior efforts to increase cancer screening in AI communities have not been reversed. Increased surveillance and monitoring of cancer-related outcomes among AI people are needed to ensure that pandemic-related delays in cancer screening do not lead to worse cancer outcomes in the AI population.
